# Walking the line: mechanisms underlying directional mRNA transport and localisation in neurons and beyond

**DOI:** 10.1007/s00018-020-03724-3

**Published:** 2020-12-20

**Authors:** Reem Abouward, Giampietro Schiavo

**Affiliations:** 1 Department of Neuromuscular Diseases, UCL Queen Square Institute of Neurology, University College London, London, WC1N 3BG UK; 2 UK Dementia Research Institute, University College London, London, WC1E 6BT UK; 3 The Francis Crick Institute, 1 Midland Rd, London, NW1 1AT UK

**Keywords:** Axonal transport, Vesicular traffic, Neurodegeneration

## Abstract

Messenger RNA (mRNA) localisation enables a high degree of spatiotemporal control on protein synthesis, which contributes to establishing the asymmetric protein distribution required to set up and maintain cellular polarity. As such, a tight control of mRNA localisation is essential for many biological processes during development and in adulthood, such as body axes determination in *Drosophila melanogaster* and synaptic plasticity in neurons. The mechanisms controlling how mRNAs are localised, including diffusion and entrapment, local degradation and directed active transport, are largely conserved across evolution and have been under investigation for decades in different biological models. In this review, we will discuss the standing of the field regarding directional mRNA transport in light of the recent discovery that RNA can hitchhike on cytoplasmic organelles, such as endolysosomes, and the impact of these transport modalities on our understanding of neuronal function during development, adulthood and in neurodegeneration.

## The biogenesis and composition of RNA granules

From their synthesis, mRNAs interact with several RNA binding proteins (RBPs) that dictate their fate, from splicing and translation to cellular localisation and degradation [1]. RBPs are recruited to mRNAs by binding to specific sequences known as *cis*-elements and/or by recognising specific secondary and/or tertiary mRNA structures [1, 2]. *Cis*-elements are scattered across the length of the mRNA, but are more frequently found within its 3′-untranslated region (3′-UTR) [3]. Such mRNA and RBP complexes are known as messenger ribonucleoprotein particles (mRNPs). Several mRNPs can come together via protein–protein and RNA-RNA interactions, forming liquid–liquid phase-separated RNA granules, such as stress granules and P-bodies [4]. The composition of the granules and the signals that trigger their formation and their subsequent functions, confer to the different RNA granules their unique identities [2]. Relevant to this review are RNA transport granules, in which mRNAs are transported in a likely translationally silent state, until they reach their targets where they undergo local translation in response to specific signals such as external spatial guidance cues [5, 6]. A general conclusion emerging from decades of research, is that the choice of where these granules go depends on a number of factors, such as the sequences of *cis*-elements in the mRNA and the protein composition of the granules. However, the full picture of how these granules are assembled or processively transported is still unavailable and is a subject of intense study [7, 8]. Nonetheless, it is clear that the transport of these granules is mediated via interactions, either direct or indirect, with the main classes of motor complexes: kinesins, cytoplasmic dynein and myosins [7, 9, 10].

## Motor proteins

Molecular motors are mechanoenzymes that hydrolyse ATP to move along cytoskeletal elements which act as a two-way railway system for moving cargo around cells. Microtubules have polarised *plus* (fast growing) and *minus* ends [11, 12], which determine microtubule orientation within cells. In axons and distal dendrites, microtubules are uniformly organised with their *plus* ends facing the axon terminal, whilst in proximal dendrites their orientation is mixed, with *plus* ends pointing in both directions [13]. Actin filaments are also polarised with *barbed* (fast growing) and *pointed* ends [14]. Motor proteins recognise the orientation of microtubules and actin microfilaments, which determines their overall direction of movement.

The kinesin and dynein superfamilies move along microtubules, with kinesins mostly moving towards the *plus* end of microtubules, and cytoplasmic dynein moving towards the *minus* end [11, 12]. In contrast, myosins travel along actin filaments, with all known myosins except myosin VI, moving towards the *barbed* end [15]. These motor proteins either directly, or through adaptor proteins, recognise and bind to various cargoes, such as cytoplasmic organelles or protein complexes, transporting them to different intracellular locations. Their roles are particularly important for long-distance transport in neurons; hence, for a complete picture of RNA transport, these proteins are briefly discussed below. However, motor proteins have been the subject of many excellent reviews [11, 12, 14, 16–19], to which we would like to direct the readers for a more in-depth discussion of their different cargoes and binding properties.

Kinesin superfamily proteins (KIFs) is a diverse family of motor proteins, encoded by 45 different genes in humans. Their structure typically consists of a globular motor domain, a stalk region and a tail domain, as exemplified by conventional kinesin, known also as KIF5 or kinesin-1 [11]. KIFs are broadly grouped into three subtypes based on the location of the motor domain within the kinesin heavy chain (KHC), and classified into 14 different classes depending on their phylogeny. They bind their cargoes through the tail region of KHC, which exhibits a high degree of sequence diversity, or through associated kinesin light chains (KLCs) and adaptor proteins. The variety of the tails and light chains, further diversified via alternative splicing, enables different kinesins to bind distinct cargoes with a high level of specificity, albeit with some degree of redundancy amongst the different KIF family members and adaptors; for example, FEZ1, a kinesin-1 adaptor, has been reported to bind mitochondria as well as synaptic vesicles [17, 20, 21].

Cytoplasmic dynein, henceforth referred to as dynein, is the motor protein responsible for most of the microtubule *minus* end-directed intracellular traffic, transporting a wide variety of membrane-bound and membrane-less cargoes [12, 19]. Structurally, the dynein transport complex is made of a dimer of dynein heavy chain (DHC), which encompasses the motor and dimerisation domains [22], and several accessory subunits, such as dynein intermediate chains (DICs), light intermediate chains (DLICs) and light chains (DLCs) [19]. In its active form, dynein is bound to the dynactin complex and a diverse set of adaptor/activator proteins, linking it to its myriad intracellular cargoes [19]. Additionally, the accessory subunits that bind to the DHC dimer are present in several isoforms, thus allowing the formation of a range of diverse dynein transport complexes, further increasing the variety and specificity of cargo binding [12, 19]

The myosin superfamily is formed of a large group of motor proteins, which in humans are classified phylogenetically into more than 20 classes, consisting of as many as 40 genes [15, 16, 23]. It contains the first discovered motor protein, myosin II, which subsequently led to the discovery of the other motor complexes. The force-generating mechanism of these molecular motors has been well-characterised in muscles, where muscle myosin II moves along actin filaments causing muscles to contract [24]. Myosins are also found in other systems, such as in the stereocilia of auditory hair cells, where they play additional structural roles [15, 25]. They are generally present as dimers of a heavy chain, made of a motor region, a neck region and a diverse tail region used to bind a variety of cargoes [24].

Several studies over the last two decades have identified binding of mRNA transport granules to motor proteins belonging to all three families [9, 26]. In some model systems, the composition of the mRNA transport complex, from the RBPs to the adaptors and associated motors, has been characterised but in most cases the full picture is still far from clear. The following sections will discuss some of the best studied models, highlighting relevant lessons and existing knowledge gaps in each, starting with simpler models and moving onto more complex systems.

### mRNA transport in budding yeast

Budding yeast can exist in two different mating types in haploid yeast cells. Following budding, the expression of a site-specific endonuclease, HO, is maintained in mother cells, allowing them to switch their mating-type [27, 28]. However, endonuclease HO expression is inhibited in daughter cells, due to the segregation of its transcriptional repressor, Ash1p, specifically into daughter cells, thereby inhibiting mating-type switching. This process ensures that the mother and daughter cells have different mating types, allowing them to fuse and form diploid nuclei under permissible conditions [27, 28]. The asymmetric distribution of Ash1p is due to the transport of its mRNA, *ASH1*, into the bud tips via an actin-dependent process during budding [29, 30].

Five independent *She* genes have been shown to be important regulators of mating-type switching [29]. Yeast cells lacking these genes showed symmetrically distributed *ASH1* mRNA across the mother and daughter cells, leading to the global repression of mating-type switching [31, 32]. Moreover, symmetrical *ASH1* distribution has also been observed upon treatment of yeast cells with the actin depolymerising drug, latrunculin, and in mutants for proteins important for actin functions, indicating that *ASH1* mRNA localisation to the yeast bud is actin-dependent [31, 32]. One of the five *She* genes identified as regulators of mating-type switching encodes She1p, which is the type-V non-processive single-headed myosin motor known as Myo4p. Immunoprecipitation studies showed that *ASH1* mRNA associates with Myo4p, but only in the presence of two other She proteins, She3p and She2p [33, 34]. She3p was able to bind Myo4p directly even in RNA degrading conditions, but it co-immunoprecipitated with *ASH1* mRNA only in the presence of She2p [34]. A separate set of studies using the yeast two-hybrid and three-hybrid systems demonstrated that She3p acted as an adaptor between She2p and Myo4p [35, 36].

In contrast, She2p was able to pull-down *ASH1* mRNA even in mutants lacking She3p and Myo4p [34]. *ASH1* mRNA contains four *cis*-elements that are important for its localisation, including one in the 3′-UTR [37, 38]. UV-crosslinking showed that She2p can bind all four localisation elements, but with different affinities, demonstrating that it is an RNA-binding protein [36]. The binding of She2p to She3p was RNA-independent, as a She2p mutant unable to bind RNA was still capable of interacting with She3p [39]. Later experiments then showed that She2p binds *ASH1* co-transcriptionally in the nucleus forming a pre-complex that is shuttled to the cytoplasm [40–42].

Structural analysis and in vitro reconstitution studies have revealed that Myo4p is present in the cytoplasm in an inactive state bound to a She3p dimer and that She2p binds *ASH1* as a tetramer [43–45]. Following its nuclear shuttling, the RNA-She2p precomplex recruits two Myo4p-She3p motor complexes, effectively coupling two single-headed myosin motors and turning them into processive motor complexes [43–45]. Recent structural analysis showed that *ASH1* undergoes marked conformational changes upon She2p binding [46]. The binding of this complex to She3p further restricts the structure of *ASH1*, increasing the specificity of She2p association with *ASH1* mRNA and enabling She3p to specifically bind to specific *ASH1* localisation elements. Crucially, these sequential changes drive the stabilisation of the forming RNP transport complex [46].

**Fig. 1:**
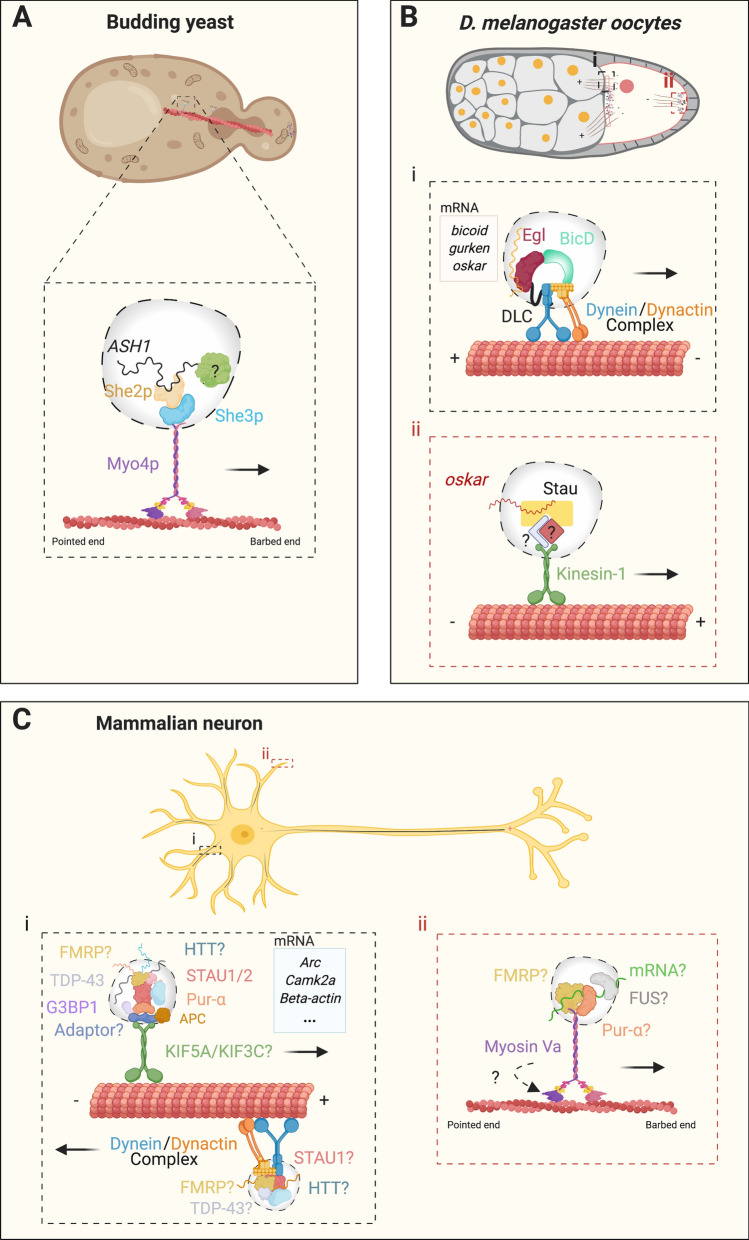
Summary of direct motor-mRNA granule interactions for mRNA transport across different systems. **a** Proteins involved in *ASH1* mRNA transport along actin filaments into the newly formed bud in budding yeast. Additional RBPs still to be identified are marked by a question mark. **b**-i Diagram of a stage 10/12 *Drosophila* oocyte, showing the Egl-BicD complex mediating the transport of *bicoid*, *gurken* and *oscar* mRNAs from nurse cells into the forming oocyte during oogenesis. *Oskar* mRNA is initially released into the oocyte and is later transported in a complex with kinesin-1 and Stau1 and possibly other still unidentified RBPs to the posterior pole (**b**-ii). **c** Schematic summarising the main motor proteins and RBPs mediating mRNA transport in dendrites and axons (**c**-i) and potentially in pre- and post-synaptic regions (**c**-ii). The adaptor identity in (**c**-i) is currently unknown, in vitro reconstitution studies suggest KAP3 to be one potential adaptor [96]. Question marks indicate that the stoichiometry and exact composition of these complexes are still unclear. Furthermore, whether myosin Va drives the transport of mRNPs is still under investigation. Ribosomal proteins have also been observed in mRNA transport granules in neurons, but have been omitted for clarity. See main text for further information

From these studies, it was concluded that *ASH1* mRNA is transported into the yeast bud in a complex made of She2p, She3p and Myo4p (Fig. 1a). In this complex, Myo4p powers the movement of the complex along actin filaments towards the *barbed* end facing the newly forming bud [28]. Interestingly, some studies have suggested that the mRNA moiety maybe be important for Myo4p processivity, as it was found that a transport complex preserving protein–protein interactions, but lacking the *ASH1* mRNA, is unable to efficiently translocate to the bud tip [39]. Indeed, artificially tethering *LacZ* mRNA to She3p was sufficient for the asymmetric distribution of She3p and Myo4p to the yeast bud [36, 40]. The aforementioned in vitro reconstitution studies [44, 45] conclusively demonstrated that the impact of mRNA binding on Myo4p processivity is at least partly due to the pairing of two Myo4p proteins by She2p. They however, reached contradicting conclusions on the role of *ASH1* mRNA itself, with one study concluding that the mRNA itself is essential for processivity under physiological conditions to stabilise the transport complex [45], whereas the other argued that the cargo mRNA is dispensable for in vitro processivity [44]. It therefore remains unclear if the RNA moiety is essential for transport in living cells [47, 48].

## mRNA transport in *Drosophila* oocytes

In the fly model *D. melanogaster,* the developmental axes are pre-determined through the transport of specific mRNAs from the nurse cells into the oocyte during early oogenesis [10, 49]*. Bicoid* mRNA, which encodes an essential morphogen, is transported to the anterior cortex of the oocyte, thus establishing the anterior pole [50]. *Gurken* mRNA, encoding a TGFɑ homologue, is also transported towards the anterior pole, but later localises antero-dorsally, specifying the dorsal pole [51]. In contrast, the *oskar* mRNA localises posteriorly for posterior axis specification and germ cell determination [51–53]. Unlike *ASH1* mRNA transport, these mRNAs are transported into the oocyte by dynein moving along microtubules, which are oriented with their *minus* ends pointing towards the oocyte and away from nurse cells, as treatment of *Drosophila* ovaries with microtubule-destabilising drugs disrupts this localisation [50, 54]. These mRNAs are transported in a complex with Egalitarian (Egl), an RBP, and the dynein-adaptor protein Bicaudal (BicD) [55–58]. This Egl-BicD complex also seems to underlie the apical localisation of *pair-rule* transcripts in the blastoderm syncytium, such as *wingless* and *hairy*, which are important for patterning of embryonic segments [56, 59]. In the case of *gurken* mRNA, the identity of these RNA-containing structures as membrane-less organelles was validated by electron microscopy [60].

The RNA binding properties of Egl were demonstrated via an elegant series of experiments by Simon Bullock’s team [61]. They incubated an ovary extract with immobilised minimal RNA localisation elements and identified Egl and BicD as the only proteins that specifically associated with these structures. However, Egl could bind a number of different localisation sequences, thus revealing that it is a promiscuous RBP [61], which might explain how it is able to transport a wide range of mRNAs [62]. In addition, Egl binds through its N-terminus to BicD, an adaptor and activator of the dynein-dynactin motor complex [63], which links the mRNP to the transport machinery [61]. Downregulation of Egl mislocalised *bicoid*, *gurken* and *oskar* transcripts in oocytes, showing that Egl is necessary for their correct localisation [64]. Recent studies reported the in vitro reconstitution of the *minus* end directed transport of mRNAs using only purified proteins of the Egl-BicD transport machinery, thus demonstrating that these proteins are not only necessary, but also sufficient for RNA transport (Fig. 1b) [65, 66]. However, additional factors might help direct the different mRNA cargoes to their correct location in vivo, such as Exu protein for early-stage *bicoid* mRNA localisation [50].

Egl binds to the DLC subunit of the dynein motor complex through its C-terminal domain [67]. Hypomorphic Egl mutants that lose their ability to bind DLC but not BicD, display defective RNA localisation, demonstrating that Egl binding is essential for the processivity of the motor [61, 67]. In vitro reconstituted RNA transport studies revealed that two Egl molecules are present in the moving RNA granule, and that the bivalent mRNA-bound configuration of Egl relieves autoinhibition of BicD and induces processive movement of dynein [66]. It was subsequently demonstrated that the binding of Egl to DLC, enables Egl dimerisation, potentially explaining why the lack of Egl-DLC binding results in defective RNA transport [68].

Although the dynein-dependent transport of Egl-BicD mRNPs is crucial for mRNA localisation in *Drosophila*, other motor proteins also contribute to this process. For example, once localised to the oocyte, *oskar* travels posteriorly, moving towards the *plus* ends of microtubules using conventional kinesin in a complex with Staufen (Stau) and other RBPs (Fig. 1b) [52, 53, 69, 70]. As a result, kinesin heavy chain-null oocytes have defective *oskar* mRNA localisation with normal *bicoid* distribution [69], and deletions of specific Stau domains also disrupt *oskar* localisation [71]. In addition, Stau was found to play a role in *bicoid* anterior localisation pattern at later stages of oocyte development [54, 71]. However, the precise molecular aspects of this mechanism are yet to be clarified.

## Neuronal mRNA localisation

Neurons are classic examples of polarised cells, with distinctive dendritic and axonal compartments carrying out unique functions that rely on their specific morphologies [3]. Indeed, their dendritic tree can be extremely complex, and their axons can extend more than a meter in length in large mammals, including humans. mRNA localisation is crucial for establishing and maintaining such a polarity [3]. Additionally, neurons host up to several thousand synapses where mRNA localisation and local translation are key for ensuring the rapid spatio-temporal regulation of protein synthesis required for synaptic plasticity [3, 6, 72]. For instance, *Camk2ɑ* mRNA was found to localise to dendrites at sites receiving high-frequency stimulation [73], whereas NMDA-induced neuronal activation was demonstrated to trigger the translocation of *Calmodulin-3* mRNA into dendrites in rat cortical neurons [74]. Deep sequencing of hippocampal neuropil coupled with Nanostring analysis and high-resolution fluorescent in situ hybridisation, estimated the number of distally localised mRNAs in dendrites to be around 2500 [75]. Imaging studies also demonstrated that mRNA translation occurs in a specific and spatially restricted manner in stimulated synapses in *Aplysia* (a sea slug) neurons, thereby mediating long-term synaptic plasticity [76]. This combined evidence clearly supports the hypothesis that mRNA localisation and translation are important phenomena in dendrites [6].

However until a few years ago, it was less widely accepted that such processes take place in axons despite early supporting evidence dating back to the 1960s [77]. Pioneering studies led by Christine Holt’s group demonstrated that growth cones in developing axons contain *beta actin* transcripts, which are translated in response to extrinsic guidance cues only in the immediate vicinity of the specific spatial signal [78]. Indeed, acute inhibition of protein synthesis in *Xenopus* larval brains disrupts axonal branching dynamics of developing axons detached from their somas in the tectum, demonstrating the in vivo importance of axonal protein synthesis during development [79]. Similar findings obtained through the immunoisolation of genetically tagged ribosomes from axons followed by deep sequencing of associated transcripts showed that thousands of axonally localised mRNAs undergo active translation in vivo, and that the axonal translatome changes dynamically during development [80, 81]. Such studies in addition to early evidence of axonal polysomes and mRNA granules, coupled with axonal bulk transcriptomics, conclusively demonstrated that mRNAs can be recruited and transported into axons where they undergo local translation to support axonal growth and maturation in both sensory and motor neurons in vitro and in vivo [77, 82–85]. mRNA transport and translation continue to play important roles in adulthood for processes such as axonal regeneration after injury [86–88]. More recently, these concepts were extended to pre- and post-synaptic regions, and local protein synthesis was found to be a common feature of both compartments [89].

## Neuronal RNA transport granules

The molecular composition of neuronal RNA transport granules is still not completely clear. However, several RBPs such as, STAU, FMRP (Fragile X Mental Retardation Protein) and G3BP1 (ras-GTPase-Activating Protein SH3-Domain-Binding Protein 1), are known to be associated with these granules [87, 90–92]. Nevertheless, how these RBPs interact with motor proteins remains undetermined. Early studies identified a 1000S complex from mouse brain homogenates as a binding partner of kinesin-1 using an immunoprecipitation approach [93]. Several RBPs, including Pur proteins, FMRP and STAU, were found associated with this complex in an RNase-insensitive manner. Reverse-transcription polymerase chain reaction (RT-PCR) demonstrated that *Camk2a* and *Arc* mRNAs were present in the immunoprecipitates, thereby suggesting that the 1000S complex is an RNA transport granule (Fig. 1c). Immunostaining experiments for Pur-ɑ, the strongest binding partner of kinesin-1 in this structure, showed a punctate distribution in dendrites of cultured hippocampal neurons, which co-localised with mRNAs, kinesin-1 and several RBPs [93]. These structures were then demonstrated to be RNA transport granules based on time-lapse experiments showing their bi-directional transport in cultured neurons. Overexpression of kinesin-1 caused an increase in the anterograde movement of these RNA granules away from the soma, whilst expression of a dominant-negative kinesin-1 mutant reduced their dendritic localisation. Consistently, knockdown of specific RBPs, such as STAU, also reduced their dendritic localisation. Mass spectrometry analysis of isolated complexes enabled the identification of additional RBPs, raising the number of protein components of these RNA granules to more than 40 [93]. This study therefore provided conclusive evidence that kinesin-1 mediates the transport of Pur-ɑ-positive RNA granules, perhaps through direct binding to the motor tail domain [93].

In line with the previous findings, STAU1- and STAU2-containing granules isolated from rat brain homogenates were also found to be enriched in kinesin heavy chain [94]. Furthermore, in another study, kinesin-1 pulldowns from extracts of mouse brain synaptosomes were enriched in *Rac1* and *Map1b* mRNAs, and contained FMRP, STAU1 and TAR DNA binding protein 43 (TDP-43) [92]. Additionally, FMRP was shown to bind neuronal KIF3C in a yeast two-hybrid screen using a human foetal brain cDNA library, suggesting that these findings could be extended to the human nervous system [95].

Recent in vitro studies have successfully reconstituted processive transport of *beta actin* and *b2B-tubulin* mRNAs via kinesin-2, as part of an RNA transport granule made of the RBP, adenomatous polyposis coli (APC) and the kinesin-2 adapter, KAP3 [96]. APC binds hundreds of mRNAs in the mouse brain, 45% of which are known to be present in axons. This binding is functionally important for localising at least some of these transcripts to axons, as blocking the interaction of APC to *b2B-tubulin* in mouse neurons, causes *b2B-tubulin* mislocalisation away from axons to the soma [97]. The in vitro reconstitution experiments revealed that APC bound to the mRNA cargo is associated to kinesin-2 via KAP3 [96]. Interestingly, APC was found to be required for the activation of motor processivity, whilst cargo mRNA enhances transport. One to three mRNAs can be transported simultaneously by one complex, and mRNAs with different APC binding sequences are transported at varying efficiencies and have different APC binding affinities. Such binding properties may fine-tune mRNA transport in vivo to potentially ensure that low-abundance mRNAs are also transported efficiently [96]. This landmark study thus demonstrates for the first time that a minimal complex consisting of a kinesin motor, an adaptor protein and an RBP, is sufficient for mediating RNP transport, shedding light on the stoichiometry and transport properties of RNA transport complexes.

Dynein was also reported to play a role in neuronal RNA transport. For instance, huntingtin (HTT), an interactor of dynein and kinesin, mutations of which cause Huntington’s disease, was co-transported with *beta actin* mRNA in a microtubule-dependent manner in rat cortical neurons [98]. In situ hybridisation and immunocytochemistry experiments demonstrated that 40% of *beta actin* mRNA in dendrites co-localised with HTT. A fraction of these structures also co-distributed with components of the dynein transport machinery, including DHC, as well as KIF5A (Fig. 1c). In a more recent study, already briefly mentioned, dynein immunoprecipitation from mouse synaptosome extracts pulled-down *Rac1* and *Map1b* mRNAs together with the RBPs STAU1, FMRP and TDP-43. Knockdown of STAU1, but not FMRP or TDP-43, reduced the association of dynein to these mRNAs, showing that it was required for their interaction [92]. Therefore, dynein plays a role in neuronal mRNA transport, but further investigation is needed to clarify the precise mechanism of motor-RNA interaction and the crosstalk with other motor proteins.

Actin filaments are highly enriched at pre- and postsynaptic regions in neurons, raising the possibility that myosinsplay a role in synaptic RNA transport [12]. In an early study, immunoprecipitates of Pur-ɑ and FMRP from rat brain homogenates were found to contain myosin Va, hinting at a potential interaction between RBPs and this motor protein [99]. These results were extended by another study which showed that human STAU associates with molecular motors from all three superfamilies, including myosins [100]. Moreover, TLS/FUS (translocated in liposarcoma/fused in sarcoma), an RBP involved in familial amyotrophic lateral sclerosis (ALS) [101], was found to bind myosin Va [102] and to translocate into dendritic spines. This shift can be disrupted by treatment with both actin- and microtubule-destabilising drugs, showing that it is both microtubule- and actin-dependent [102]. Interestingly, the expression of a dominant-negative form of myosin Va and its downregulation, suppressed the translocation of FUS into dendritic spines, causing its accumulation in dendritic shafts [102]. Since FUS was still transported into dendrites, myosin Va may be specifically involved in synaptic mRNP localisation (Fig. 1c). FUS was also found in the 1000S granule isolated by the Hirokawa team [93], therefore, one likely scenario that emerges is that FUS transport into dendrites is mediated via KIF5A, whilst its translocation into spines instead relies on myosin Va.

Collectively, these studies provide important insights as to which motors might be involved in mRNA transport in mammalian neurons, and confirm the identity of some of the motor complexes previously identified in lower organisms, which have been summarised in Fig. 1. However, many questions still remain unanswered. Innovative approaches, such as proximity labelling followed by mass spectrometry and RNA sequencing [103, 104] might shed light on how the binding of RNA transport granules to motors takes place in vivo and under which physiological conditions (e.g., synaptic stimulation or silencing), thus validating in vitro findings.

Crucially, an independent mRNA transport mechanism was recently discovered in the filamentous fungi and plant pathogen, *Ustilago maydis*, whereby RNA transport granules ‘hitchhike’ a ride on endosomes [105]. This concept is explored in the following sections.

## Hitchhiking onto endosomes as a mechanism of transport

To establish pathogenicity, *U. maydis* switches from a yeast-like morphology to growing unipolar hyphae, which allow this organism to invade the host plant epidermis. The hyphae are highly polarised and require directional transport of organelles, proteins and mRNA granules down their length, to support their growth and establish their polarity [106, 107]. Similarly to mammalian cells, the intracellular transport of organelles in filamentous fungi also depends on motor proteins [108, 109]. It was generally assumed that organelles are transported in fungi by the direct recruitment of motor protein complexes to their membranes; however, a recent study challenges this view [110].

The Steinberg group discovered that peroxisomes and to a lesser extent, lipid droplets (LDs) and the endoplasmic reticulum (ER), ‘piggy-back’ onto endosomes for their long-distance transport in hyphae. Peroxisome motility is dependent on dynein, kinesin-3 and the integrity of microtubules, whose *plus* ends point towards the hyphal tips. Upon simultaneous imaging of kinesin-3 and peroxisomes, the authors discovered that this motor protein takes a ‘lead’ during transport. As kinesin-3 transports endosomes in *U.maydis*, they investigated whether peroxisome transport is related to endosomal transport. When endosomes and peroxisomes were imaged in living hyphae, the authors found that these organelles co-traffic, with endosomes in the lead, suggesting that peroxisome motility might be tied to that of endosomes [110]. Indeed, when they disrupted endosomal motility by deleting the endosome-specific motor adaptor, *hok1*, peroxisome transport was completely abolished. Restoring endosome motility also restored that of peroxisomes, further supporting the notion that peroxisomes hitchhike on endosomes via a novel mechanism. Similarly, the frequency of transport of LDs and most of ER’s was also abolished in *hok1*-null hyphae [110]. Interestingly, the PdxA protein was identified as a linker between endosomes and peroxisomes in *Aspergillus nidulans*, another filamentous fungus, where co-transport of peroxisomes and endosomes also occurs [111].

### mRNA hitchhiking in filamentous fungi

**Fig. 2:**
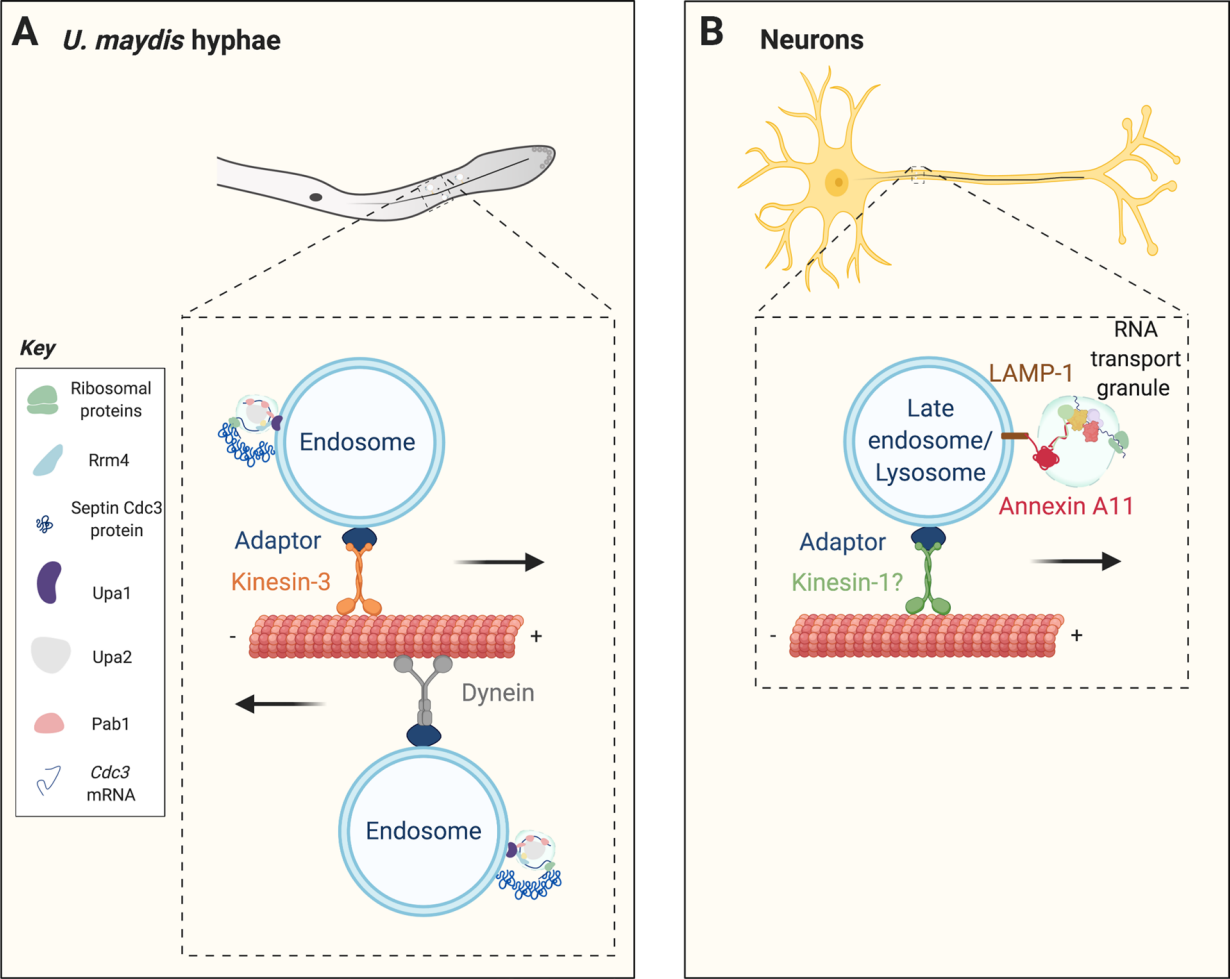
Organelle hitchhiking as a mechanism of mRNA granule transport. **a** Schematic showing the machinery involved in the transport of *cdc3* mRNA in *U. maydis*. All four septin mRNAs and the corresponding proteins have also been shown to localise to shuttling endosomes, but they have been omitted for clarity. Pab1 stands for poly-A binding protein. Diagram of RNA granule is adapted from [116]. **b** Endosomal/lysosomal hitchhiking in neurons. ANXA11 acts as a tether between RNA granules and LAMP1-positive organelles by exploiting a phase-separation mechanism only partially understood. G3BP1 is one of the components of these transported RNA granules. See main text for additional details

The findings described above also extend to mRNA transport, where several studies have unveiled a novel mechanism whereby mRNAs hitchhike onto endosomes, exploiting them for transport. Rrm4 is an RNA-binding protein in *U. maydi*s that was shown to be essential for microtubule-dependent transport of mRNAs [112]. For example, Rrm4-mediated transport of the endochitinase *cts1* mRNA to the hyphal growth cone is required for the secretion of Cts1 protein, which is important for maintaining the integrity of the hyphal cell wall, thus demonstrating the functional relevance of mRNA transport in filamentous fungi [113]. Rrm4 was found to co-localise with endosomes, and the transport of Rrm4-associated mRNPs required functional endosomes [105] (Fig. 2a). The authors of this study used a strain of *U. maydis* carrying a temperature-sensitive *yup1* allele encoding the endosomal t-SNARE Yup1, which under restrictive conditions displays impaired endosomal dynamics [105]. In this strain, Rrm4 shuttled normally at permissive temperatures, but processive movement was hardly observed after switching to restrictive conditions, thus demonstrating the dependence of Rrm4-mediated mRNP transport on endosomes. In addition, endosomal mobility was found to be required for the homogeneous distribution of polysomes within hyphal cells [114]. More recent experiments revealed that Rrm4 associates with endosomes via an intermediate protein, Upa1 [115]. Upa1 binds endosomes through a C-terminal FYVE zinc-finger domain (known to bind phosphoinositides), and binds Rrm4 through other regions involved in protein–protein interactions. Upa1 deletion mutants lack processive Rrm4 movement, display impaired mRNA localisation and defective hyphal growth [115]. Another protein, Upa2, was also found to be important for RNA transport in hyphae. Its deletion causes a 50% reduction in the number of processively-moving mRNAs and causes defects in hyphal growth. This protein is thought to act as a scaffold stabilising the RNA granule complex during transport [116].

An additional role for endosomes as mRNA translation platforms for septin *cdc3* mRNA was recently identified [107]. The Feldbrügge group demonstrated that the distribution of septin Cdc3 to the hyphal growth cone and septa is lost in Rrm4-null cells. Live imaging of fluorescently tagged *cdc3* mRNA and Cdc3 protein showed that they localise to shuttling endosomes, and further mutational studies revealed that the association of Cdc3 protein with endosomes is Rrm4-dependent (Fig. 2a). Ribosomal proteins and another septin family member, Cdc12, were also recruited to Rrm4-positive shuttling endosomes [107]. A follow-up study revealed that all four septin mRNAs and proteins are found on endosomes, again in an Rrm4-dependent manner [117]. Interestingly, Cdc3 and Cdc12 proteins were targeted to the same subcellular locations and their co-localisation was strongly reduced in the absence of Rrm4. Altogether, these findings suggest that endosomes are acting as translation hubs for septin mRNAs, whereby the close spatial association of newly synthesised septins on endosomes facilitates their co-assembly into heterooligomeric complexes, and their subsequent delivery to target locations [107]. In this case, mRNA transport seems to drive not only asymmetric localisation of mRNAs to support site-specific protein synthesis, but may also play additional roles, such as enabling the efficient assembly of protein complexes in situ. Such a hypothesis might explain why mRNAs undergo bidirectional shuttling in axons [118].

From these studies, it emerges that endosomes act as a general platform for intracellular transport in filamentous fungi, enabling the transport of proteins, lipids, mRNPs and various organelles over long distances [108]. A deeper look through the budding yeast literature suggests that *ASH1* mRNA might also be hitchhiking onto membrane-bound organelles for transport, and that heterooligomeric complexes are also assembled co-translationally but it is not clear whether such translation is related to endosomes or other membrane-bound organelles [119, 120].

Efforts investigating whether this mechanism of mRNA transport and translation extends to higher eukaryotes have only just started, with a number of recently published papers demonstrating associations of mRNA transcripts with endolysosomal compartments in neurons and their potential co-trafficking [91, 121].

### mRNA hitchhiking in neurons

Recently, the Ward team set out to investigate whether mRNA granules are transported by hitchhiking onto motile organelles [91]. To this end, they heat-shocked human bone osteosarcoma U2OS cells expressing mCherry-G3BP1, inducing the formation of stress granules. G3BP1 is an RBP involved in the formation of RNA stress granules [122]. By simultaneously tracking the movement of several organelles and G3BP1-labelled structures, the authors discovered that stress granules co-trafficked with LAMP1-positive late-endosomes/lysosomes (Fig. 2b). Correlative light-electron microscopy imaging further demonstrated that stress granules were not engulfed by endolysosomes, but were juxtaposed to their delimiting membrane. In cultured primary rat cortical neurons, which constitutively transport RNPs within their axons, *beta actin* mRNA labelled with the MS2/MCP system [123] co-trafficked with LAMP1-positive lysosomes, validating the results obtained in U2OS cells.

To elucidate the mechanism underlying the interaction between lysosomes and mRNA granules, they employed ascorbate peroxidase (APEX) proximity labelling proteomics using LAMP1 as a bait in non-heat shocked neurons derived from human induced pluripotent stem cells. This LAMP1 interactome was cross-referenced with a separate APEX study that used G3BP1 as a bait, to identify proteins interacting with both lysosomes and G3BP1-positive RNA stress granules. Through this approach, annexin A11 (ANXA11), a member of the annexin superfamily of scaffolding proteins, was identified [91].

Follow-up imaging studies showed that ANXA11 is recruited to stress granules in U2OS cells, and that it is co-trafficked with LAMP1-positive organelles in primary mammalian neurons and in vivo in zebrafish axons [91]. Additionally, ANXA11 was sufficient to induce binding of purified RNA granule cores to liposomes in the presence of calcium. Further characterisation of ANXA11′s biophysical properties showed that it contains a highly disordered N-terminal domain facilitating phase-separation in vitro and in U2OS cells. Furthermore, ANXA11 binds in a calcium-dependent manner negatively-charged phosphatidylinositol lipids, which are enriched in the membrane of late endosome/lysosome [91]. These properties provide the basis on how ANXA11 may act as a tether between RNA granules and LAMP1-positive organelles (Fig. 2b). Supporting these findings, knockdown of ANXA11 in primary neurons reduces the co-transport of LAMP1-positive late endosome/lysosomes and *beta actin* mRNA, as well as the number of *beta actin* transcripts at growth cones.

Interestingly, ALS-causing mutations in ANXA11 significantly reduce its association with LAMP1-positive compartments in primary neurons, and alter its phase-separation properties, promoting the formation of more stable and possibly aggregation-prone RNA granules [91]. However, the effect of these mutations was less striking in zebrafish neurons in vivo. A likely explanation of this finding is that ANXA11 is just one of the many tethers linking mRNA granules and LAMP1-positive compartments in zebrafish, since it only contributed a small percentage to the overall transport of other types of mRNA granules (e.g., containing the RBP caprin) in this model system [91]. As such, ANXA11-mediated hitchhiking is unlikely to be the only mechanism mediating mRNA transport in neurons. Nonetheless, this study demonstrated that RNA hitchhiking is a physiological strategy for axonal mRNA transport and that this process might be impaired in ALS.

Independently, the Holt group investigated a similar process in *Xenopus* retinal ganglion cell (RGC) axons [121]. Using a fluorescently tagged uridine-5′-triphosphate, they labelled all newly synthesized RNAs in RGC axons and found that RNAs were often associated with endocytic compartments containing the small GTPases Rab5a or Rab7a. They found that 20–30% of the RNAs that colocalised with endosomes displayed bidirectional transport, and that a number of ribosomal proteins and RBPs (including Fragile X-related, which is in the same family as FMRP [124]) were also associated with endosomes [121], thus suggesting that these organelles could act as mRNA translation hubs. They pulse-labelled RGCs with a low concentration of puromycin, which mimics tRNA and incorporates into the C-terminus of newly-synthesised peptides, forming puromycylated peptides that are then released from ribosomes. This allows nascent protein synthesis to be visualised using anti-puromycin antibodies. These authors found puromycin signals associated with Rab7a-positive endosomes, which decreased if Rab7a was mutated or upon pharmacological disruption of endosomal maturation. Therefore, endosomes may act as translation hubs for mRNAs, a physiological function that could be evolutionarily conserved as it is in line with septin *cdc3* translation on endosomes in *U. maydis* [107, 117]. However, RNA transport kinetics were not altered by the expression of dominant-negative or constitutively active GTPase mutants of Rab5a or Rab7a, arguing that endosomal hitchhiking may not be essential for mRNA transport.

Interestingly, Rab7a-positive endosomes were found to often pause when they encounter mitochondria, forming apparent contacts with them that persist for over 2 min [121]. Quantitatively, around 35% of Rab7a-positive endosomes were in close proximity to mitochondria, of which 80% carried mRNA, and 76% carried a significant puromycin signal [121]. Therefore, endosomes may be facilitating mitochondrial protein synthesis, an important finding which was further substantiated by the discovery that mutations in Rab7a underlying the axonal neuropathy Charcot-Marie-Tooth disease type 2B (CMT2B), disrupt mRNA translation on endosomes, causing a parallel impairment of mitochondrial function [121]. As such, these findings raise important questions about the potential pathological consequences of endosomal hitchhiking deficits and their relevance in neurodegenerative disorders.

Consistently with the observation that ANXA11-mediated RNA hitchhiking underlies only a small proportion of RNA transport in zebrafish axons [91], endosomal hitchhiking in RGC axons does not seem to be strictly required to traffic RNA granules [121]. In light of these findings, it would be important to extend the co-trafficking assays used in these studies to ascertain which organelles mediate RNA hitchhiking in neurons and whether this is affected by specific conditions, such as cellular stress. Nevertheless, these pioneering studies strongly indicate that RNA hitchhiking occurs in axons and that this process might represent an evolutionary conserved mechanism enabling directional mRNA transport and localisation.

### A potential unifying pathomechanism for neurodegenerative disorders

Defects in RNA processing such as translation, splicing and transport have been documented in several neurodegenerative disorders such as, ALS, frontotemporal dementia (FTD) and Huntington's disease [125, 126]. A prominent feature of such disorders is the formation of nuclear and cytoplasmic RNP aggregates; for instance, TDP-43 aggregates are commonly observed in ALS and FTD regardless of the underlying genetic cause [125, 127, 128]. These aggregates could further impair RNA processing by sequestering RNAs and RBPs, thus preventing them from carrying out their functions. Aggregates are thought to arise from alterations in the assembly, disassembly and/or clearance of endogenous RNA granules, such as stress granules [122, 125]. As their name suggests, stress granules are liquid–liquid phase-separated RNA granules that accumulate in neurons exposed to environmental stress. These granules isolate cytoplasmic mRNAs mostly in a translationally silent state and contain RBPs such as the translational repressors FMRP and G3BP1, and proteins involved in proteostasis, such as chaperones for protein folding and autophagy factors [129, 130].

Genetic studies have revealed that mutations in many of the RBPs found in stress granules are linked to neurological disorders; it is indeed possible that such mutations might alter the phase-separation properties of stress granules in a manner that increases their propensity to form even in the absence of stress, or making them more stable, or resistant to degradation [125]. This might facilitate the transition of stress granules from phase-separated, soluble RNA granules into insoluble and potentially toxic, amyloid-like aggregates, which in turn may impair many neuronal processes, and trigger apoptosis [125]. These changes in mRNA dynamics are currently investigated as pathogenic mediators of ALS and FTD, since mutations in several RBPs were found to be genetically associated with these disorders [127], as well as, in Fragile X syndrome which is caused by loss-of-function mutations in FMRP [131]. In addition to changes in stress granule dynamics, mouse models of ALS also demonstrated that mislocalisation of RBPs and changes in their splicing activities are sufficient to cause motor neuron degeneration, without nuclear depletion and cytoplasmic aggregation, such as in the humanised mouse model of FUS-ALS [132] and a TDP-43 gain-of-function ALS model [133]. Therefore, the homeostatic control of RNA metabolism is crucial for neuronal health.

Axonal transport dyshomeostasis is another feature seen in most, if not all, neurodegenerative disorders, including hereditary spastic paraplegias (HSPs), Huntington's disease and ALS [134, 135]. While it is not completely clear whether these axonal transport deficits represent a cause or consequence of the underlying pathology [18, 135], their early appearance prior to symptom onset in animal models of neurodegeneration suggests they are likely to play a causative role in the pathogenesis of these disorders [135]. For instance, mutations in KIF1A cause a range of phenotypes including HSP and CMT, whereas its loss in humans and mice disrupts neurotrophic signalling in sensory neurons leading to sensory neuropathy [135]. More recently, mutations in KIF5A have been linked to ALS through genome-wide analysis [136]. Additionally, mutations in the retrograde transport machinery have also been linked to neurodegenerative disorders as in the case of dynactin, whose mutations were found to cause distal hereditary motor neuropathy type 7 [135]. Moreover, it is becoming increasingly recognised that axonal mRNA transport is also disrupted in neurodegenerative disorders. For example, TDP43-associated RNA granules traffic bidirectionally in neurons [92, 137]. However, their motility is significantly impaired in neurons made from human induced pluripotent stem cells derived from patients carrying ALS-associated TDP-43 mutations [137].

Despite decades of research into the mechanisms causing neurodegeneration, a major gap in our understanding lies in how axonal transport deficits are tied to the concomitant transcriptional and translational changes taking place during the progression of neurodegenerative disorders. The aforementioned findings by the Ward and Holt groups [91, 121] that mutations linked to ALS and CMT2B disrupt the coupling of mRNAs to endosomes and their translation on these organelles, may finally enable us to bridge together these two fields of research. These findings raise the possibility that disrupted organelle transport simultaneously impairs axonal and dendritic RNA transport and translation [138]. RNA and RBP concentration and location are critical factors for the regulation of RNA dynamics, including phase-separation [4, 139]. Therefore, we could envisage a likely scenario whereby RNA transport deficits mislocalise RNA granules and alter the RNA and RBP levels in the somatodendritic and axonal compartments, in a manner that impairs RNA processing and/or promotes the formation of RNA granules, such as stress granules and P-bodies. Abnormal granule formation could in turn sequester RBPs in the cytoplasm, potentially preventing them from carrying out their physiological functions elsewhere, for example in the nucleus, as has been proposed for TDP-43 in ALS [140]. Additionally, a decline in RNA transport to axons and dendrites would disrupt homeostasis of the distal neuronal translatome and proteome, which coupled with impaired organelle transport, would further exacerbate neuronal damage. In this novel pathological framework, mRNA dynamics and organelle transport regulation are tightly coupled, offering new ways of interpreting previous findings, and paving the way for new potential therapeutic interventions acting simultaneously on both aspects of this fascinating transport and localisation pathway [138].

## Future perspectives

The findings by the Holt and Ward groups provide strong evidence that hitchhiking on endolysosomal organelles could be an evolutionarily conserved mRNA transport mechanism, with intriguing roles in homeostatic regulation of the nervous system. Their findings are bolstered by discoveries that micro-RNAs and some components of the mRNA degradation machinery seem to be transported by late endosomes/lysosomes in dendrites and axons, and that they often stall next to mitochondria at axonal branch points [141–143].

These studies raise other intriguing questions about potential interplay between such non-canonical roles of endosomes with their more established functions in regulating growth factor signalling. A subset of endosomes known as signalling endosomes, travel back to the cell body from axon terminals carrying neurotrophic factors and their activated receptors, a process that modulates gene expression affecting neuronal survival and branching [144, 145]. A recent study performed in non-neuronal cell types showed that endosomes carry mRNAs encoding proteins involved in regulating endosomal fusion and trafficking, such as *EEA1* [146]. *EEA1* transcripts are bound on early endosomes by several proteins, one of which is CSRP1, a transcriptional regulator that represses *EEA1* endosomal translation. Although EEA1-positive endosomes are largely lacking in axons [147], such findings raise the possibility that endosomes may be able to regulate their own composition locally and independently of the cell body, allowing them to rapidly modulate growth factor signalling in a spatiotemporal-specific manner. Endolysosomal hitchhiking is thus increasingly becoming a relevant mechanism for directional RNA transport and for local regulation of mRNA processing, for example in translation and degradation, with wider implications on the health of the nervous system that need future investigation.

Many questions and gaps are left to be answered in the field of RNA hitchhiking. For instance, which organelles/states are involved in mRNA hitchhiking and how do the dynamic contact sites forming between organelles contribute to this process? Additionally, we ought to characterise the components that mediate hitchhiking of RNA granules onto different organelles, such as understanding which linker proteins and RBPs are involved. Moreover, it is unclear whether certain transcripts are especially trafficked by this mechanism of transport and what specific roles this might play in regulating homeostasis within the distal neuronal compartments. It is also important to ask whether this mechanism of transport contributes in a precise manner to neurodegenerative diseases.

A large body of evidence shows that mRNA granules are transported by direct binding to motor proteins. Therefore, another important question that arises is how this mode of transport integrates with organelle-based mRNA hitchhiking. To date, there have been no studies investigating how these two mechanisms act as parallel routes for mRNA transport. They may, for instance, carry different mRNA cargoes to distinct subcellular locations, or perhaps they may carry overlapping sets of transcripts, but are preferentially deployed during specific cellular states, such as stress. These distinct transport mechanisms may also transport mRNAs to different compartments within neurons, for example, direct motor-mRNP interactions could be used for dendritic/short-distance transport of mRNA granules, whilst mRNA hitchhiking could be employed for long-distance transport down the axon. Further studies are urgently needed to test these hypotheses and explore whether such mechanisms are complementary or mutually exclusive.

## Concluding remarks

mRNA transport is a conserved phenomenon that is essential for the correct development and functioning of many organisms across the evolutionary landscape. Major strides have been made in our understanding of mRNA transport from studies in animal models and tissues investigating seemingly unrelated processes. However, despite the vast knowledge acquired so far in mRNA transport dynamics, a new and evolutionary conserved pathway of mRNA transport via organelle hitchhiking has now emerged. Endolysosomal hitchhiking may be acting as a parsimonious mechanism for conserving cellular energy by tying the transport of organelles and mRNAs to one “carrier” system. This mechanism might also be playing important roles in grouping specific mRNAs together for efficient targeting to certain organelles, such as the mitochondria. Whatever maybe the case, there are many exciting questions that are waiting to be addressed, perhaps ushering a new era of axonal transport research.
